# Astrocyte Mitochondria Are a Sensitive Target of PCB52 and its Human-Relevant Metabolites

**DOI:** 10.1021/acschemneuro.4c00116

**Published:** 2024-07-02

**Authors:** Neha Paranjape, Stefan Strack, Hans-Joachim Lehmler, Jonathan A. Doorn

**Affiliations:** † Department of Pharmaceutical Sciences & Experimental Therapeutics, College of Pharmacy, University of Iowa, Iowa City, Iowa 52242, United States; ‡ Department of Neuroscience and Pharmacology, University of Iowa Carver College of Medicine, Iowa City, Iowa 52242, United States; § Department of Occupational and Environmental Health, College of Public Health, University of Iowa, Iowa City, Iowa 52242, United States

**Keywords:** polychlorinated biphenyls, astrocytes, mitochondria, PCB metabolites, neurotoxicity, mechanisms

## Abstract

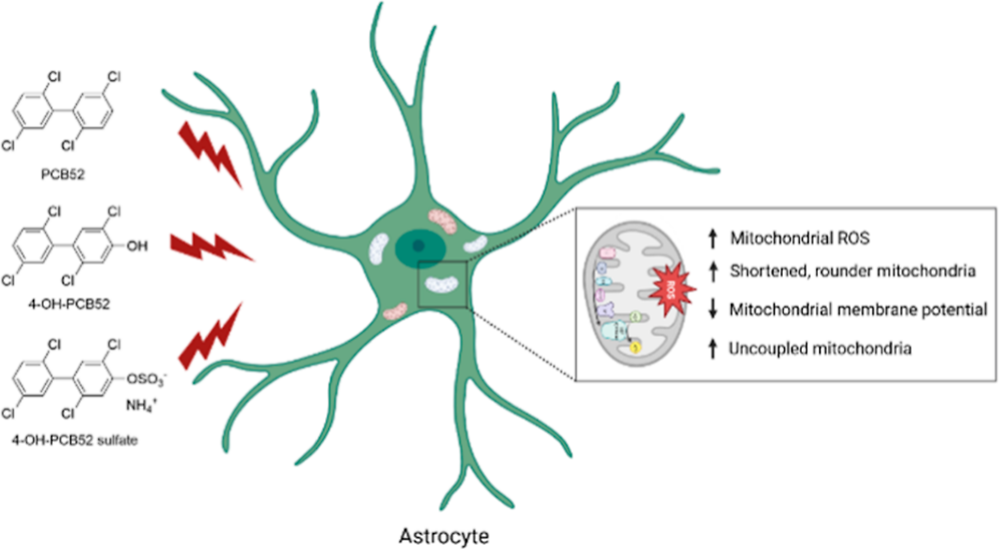

Polychlorinated biphenyls (PCBs) are industrial chemicals that are ubiquitously found in the environment. Exposure to these compounds has been associated with neurotoxic outcomes; however, the underlying mechanisms for such outcomes remain to be fully understood. Recent studies have shown that astrocytes, the most abundant glial cell type in the brain, are susceptible to PCB exposure as well as exposure to human-relevant metabolites of PCBs. Astrocytes are critical for maintaining healthy brain function due to their unique functional attributes and positioning within the neuronal networks in the brain. In this study, we assessed the toxicity of PCB52, one of the most abundantly found PCB congeners in outdoor and indoor air, and two of its human-relevant metabolites, on astrocyte mitochondria. We exposed C6 cells, an astrocyte cell line, to PCB52 or its human-relevant metabolites and found that all the compounds showed increased toxicity in galactose-containing media compared to that in the glucose-containing media, indicating the involvement of mitochondria in observed toxicity. Additionally, we also found increased oxidative stress upon exposure to PCB52 metabolites. All three compounds caused a loss of mitochondrial membrane potential, distinct changes in the mitochondrial structure, and impaired mitochondrial function. The hydroxylated metabolite 4-OH-PCB52 likely functions as an uncoupler of mitochondria. This is the first study to report the adverse effects of exposure to PCB52 and its human-relevant metabolites on the mitochondrial structure and function in astrocytes.

## Introduction

Astrocytes are macroglial cells and are the most abundant glial cell type in the central nervous system (CNS).^1^ Initially thought to provide just physical support to neurons, astrocytes are now known to perform a myriad of functions that are critical in maintaining neuron health as well as the development of diseases of the CNS.^2,3^ Some of the functions of astrocytes include maintenance of the blood–brain barrier, synapse pruning and regulation, maintenance of ion and neurotransmitter homeostasis, and regulation of energy homeostasis.^4^ Astrocytes, along with other cell types of the brain, such as microglia, can become reactive under a particular stimulus and differentiate into populations that exhibit neurotoxic and neuroprotective phenotypes.^5^ Astrocyte mitochondria are necessary to perform functions of astrocytes such as the transfer of energy-rich substrates like lactate to the neurons, Ca^2+^ signaling, and neurotransmitter uptake and recycling.^6,7^ In addition, neurons transfer their damaged mitochondria to astrocytes for repair and/or recycling, while astrocytes shuttle their healthy mitochondria to neurons to meet their high energy demand or improve neuronal health.^8,9^ Exposure to environmental pollutants can disrupt this astrocyte–neuron interaction and function, which is implicated in the development of adverse outcomes of the nervous system, such as the neurodevelopmental and neurodegenerative conditions.^5,10^

Polychlorinated biphenyls (PCBs) are persistent organic pollutants previously used for various industrial applications, such as transformers and capacitors, hydraulic fluids, window caulking and sealants, and plasticizers.^3^ Despite the ban on commercial manufacturing of PCBs in the US since 1979, PCBs continue to be produced as byproducts of industrial processes such as paint and pigment manufacturing.^2,3^ PCBs are associated with varied adverse health effects, including neurodevelopmental disorders such as attention deficit hyperactivity disorder (ADHD), autism spectrum disorder (ASD), and cognitive deficits.^11,12^ PCB exposure has also been associated with neurodegenerative disorders such as the Parkinson’s disease (PD).^6,7^

Even though the association of PCB exposure and learning deficiency in children was reported as early as 1968,^13^ the mechanisms underlying these observed effects are still unknown. Previous work has focused on neurons to study PCB-induced neurotoxicity. However, the role of other major cell types in the brain, such as astrocytes, has not been investigated. Due to the vital functions of astrocytes in the CNS, it is necessary to evaluate the effects of PCBs on astrocyte health and function. Several recent studies have explored the effects of PCBs on astrocytes and the role of astrocytes in PCB-induced neurotoxicity.^14−16^ Previously, we have shown that the rat astroglial C6 cell line and primary astrocytes from rats and mice are susceptible to adverse effects via PCB exposure.^16^ PCB52 is an ortho-substituted, nondioxin-like, tetra-chlorinated biphenyl compound that is one of the most abundantly found PCB congeners in air samples contaminated with PCBs.^17,18^ While previous work established the toxicity of PCB52 and its human-relevant metabolites for C6 cells and primary astrocytes, the cellular targets yielding the adverse outcomes were not identified.

**Figure 1:**
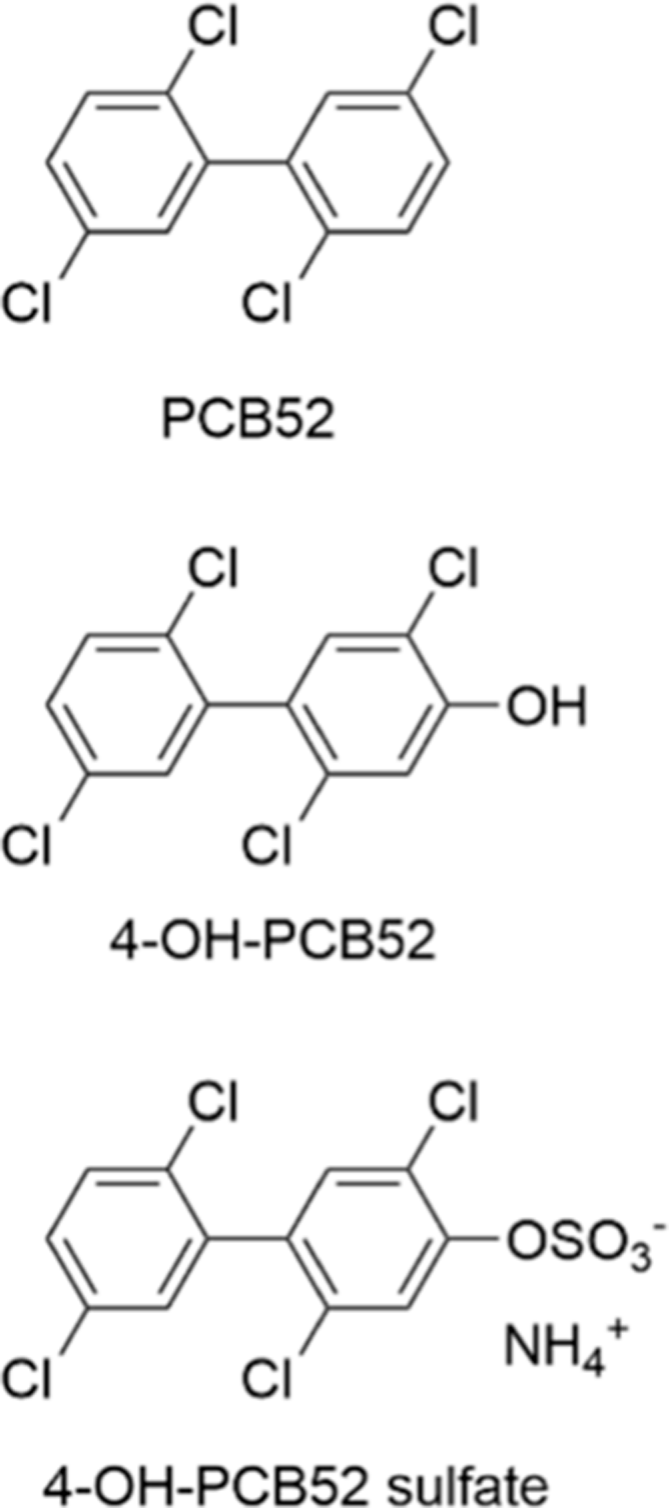
Structures of the three test compounds used in this study: PCB52, 4-OH-PCB52, and 4-OH-PCB52 sulfate.

In the present study, we investigated the unique interaction of PCB52 and its human-relevant metabolites (Figure 1) with astrocyte mitochondria as a mechanistic target. Our novel findings demonstrate that both the structure and function of mitochondria are adversely impacted via PCB52 and its human-relevant metabolites, and the outcomes are distinct depending on the chemical structure of the toxicant Figure 1.

## Results

### Culturing Cells in a Medium including Galactose rather than Glucose Shifts the IC_50_ Values for PCB52, 4-OH-PCB52, and 4-OH-PCB52 Sulfate

**Figure 2:**
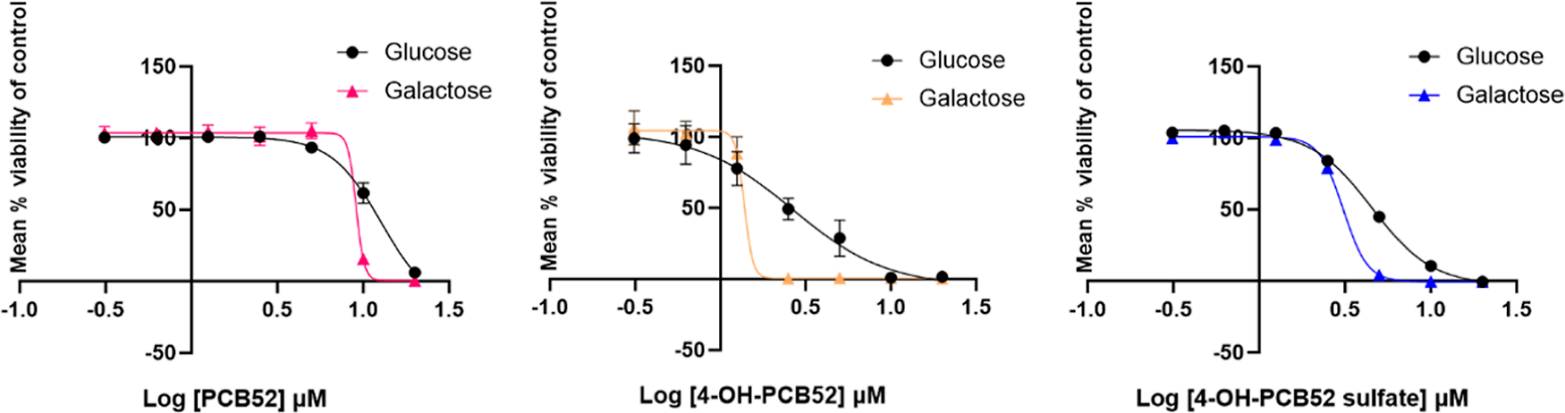
Concentration–response curves of C6 cells exposed to PCB52, 4-OH-PCB52, and 4-OH-PCB52 sulfate for 24 h at a concentration range of 0.312 to 20 μM in either glucose- or galactose-containing media. *n* = 3, data represented as mean ± SEM.

Astrocytes are primarily glycolytic under normal physiological conditions, similar to C6 astroglioma cells.^19−21^ Since galactose provides a lower yield of ATP through glycolysis than glucose, culturing cells in galactose instead of glucose will shift energy production from glycolysis to mitochondrial oxidative phosphorylation (OXPHOS).^22,23^ OXPHOS occurs in mitochondria and is a process of generating adenosine triphosphate (ATP) through a series of sequential oxidation–reduction reactions.^24^ Therefore, the glucose–galactose cell viability assay has been used to determine whether the toxicity of the test compounds depends on mitochondrial function, when the cells are exposed to the test compounds in galactose- compared to glucose-containing media.^22^ We assessed the cell viability of C6 cells exposed to PCB52 and its human-relevant metabolites in a cell culture medium prepared with either galactose or glucose. C6 cells were exposed to a concentration range of 0.312 to 20 μM of each compound for 24 h, after which the viability was assessed using the Alamar blue assay (Figure 2). The bar graphs for this cell viability assay are shown in Figure S1.

Analysis of the cell viability data demonstrates that the presence of galactose decreases the IC_50_ concentrations of all three compounds, as shown in Table 1, indicating that cellular reliance on OXPHOS increases toxicity by PCB52, 4-OH-PCB52, and 4-OH-PCB52 sulfate.

**Table 1:** Comparison of IC_50_ Concentrations of C6 Cells Exposed to PCB52 and its Human-Relevant Metabolites when Exposed to Glucose- or Galactose-Containing Media

compound tested	IC_50_ glucose (μM)	IC_50_ galactose (μM)
PCB52	12.7	9.1
4-OH-PCB52	2.6	1.4
4-OH-PCB52 sulfate	4.5	3.1

### PCB52 Metabolites Increase Mitochondrial Oxidative Stress

**Figure 3:**
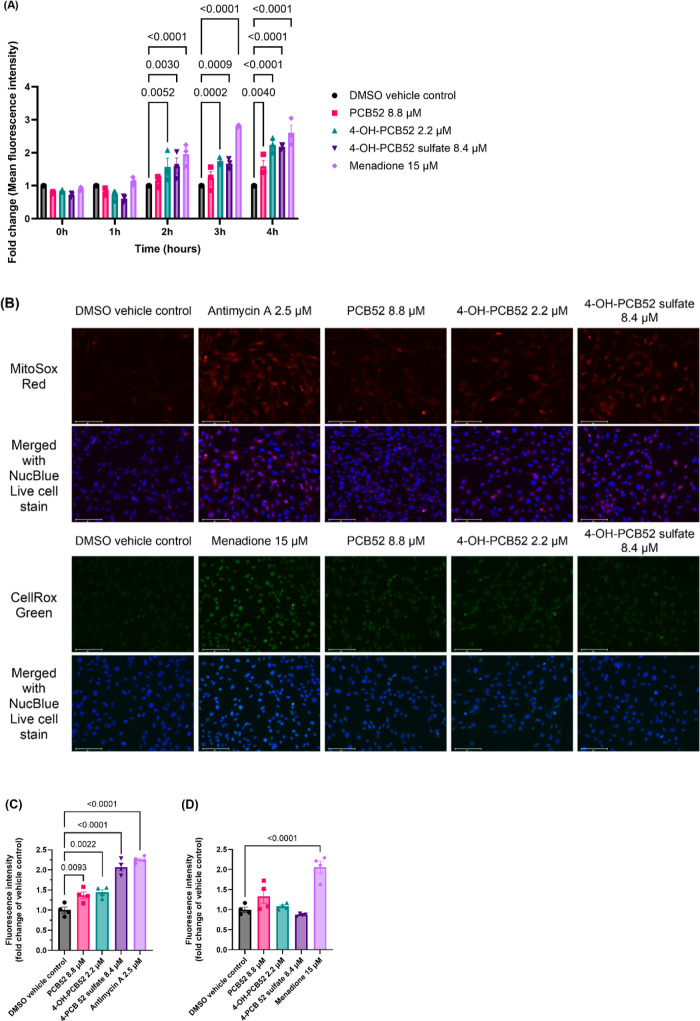
PCB52 metabolites induce mitochondria-targeted ROS. (A) Fold change of mean fluorescence intensity of DCFDA treated cells exposed to IC_50_ concentrations of PCB52 and its human-relevant metabolites. *n* = 3, with 4 technical replicates per “*n*”. (B) Live cell images of C6 cells exposed to IC_50_ concentrations of PCB52 and its human-relevant metabolites 2 h postexposure. (C) MitoSox Red and (D) CellRox Green fluorescence intensity quantification. All three compounds showed increased fluorescence for MitoSox Red but not for CellRox Green, indicating that the test compounds increased mitochondrial ROS but not cellular ROS. Scale bar, 100 μm, *n* = 4, with at least 5 images per “*n*”. Data are represented as mean ± SEM; one-way ANOVA with post hoc Dunnett’s test was used for data analysis, *p* values <0.05 indicating statistical significance are shown in the figure.

We analyzed whether PCB52 and its human-relevant metabolites increase oxidative stress in C6 cells. The cells treated with 2′,7′-dichlorofluorescin diacetate (DCFDA) and exposed to the respective IC_50_ concentrations of PCB52, 4-OH-PCB52, or 4-OH-PCB52 sulfate, and fluorescence intensity over time was measured. We found that starting 2 h postexposure, 4-OH-PCB52 and 4-OH-PCB52 sulfate significantly increased the fluorescence intensity, indicating increased oxidative stress in C6 cells (Figure 3A). We further assessed the generation of reactive oxygen species (ROS) using fluorogenic probes, MitoSox Red and CellRox Green, to detect mitochondrial and cellular ROS, respectively. C6 cells were stained with 2 μM MitoSox Red or 5 μM CellRox Green and then exposed to PCB52, 4-OH-PCB52, or 4-OH-PCB52 sulfate at their respective IC_50_ concentrations. Two hours postexposure, PCB52 and its human-relevant metabolites showed increased mitochondrial oxidative stress but not cellular oxidative stress (Figure 3B–D), as seen from the fluorescence microscopy images in Figure 3B that were further quantified for fluorescence intensity as shown in Figure 3C,D. Surprisingly, pretreatment of C6 cells for 2 h with mitochondria-targeted antioxidants, Mito-Tempo and Mito-Q,^25^ did not show protection against cell viability (Figure S2). These findings suggest that PCB52 and its metabolites increase mitochondrial oxidative stress in C6 cells. However, the increased number of mitochondrial ROS may not be the driving factor for observed cell death in C6 cells.

### PCB52 and its Human-Relevant Metabolites Cause Loss of Mitochondrial Membrane Potential in C6 Cells

**Figure 4:**
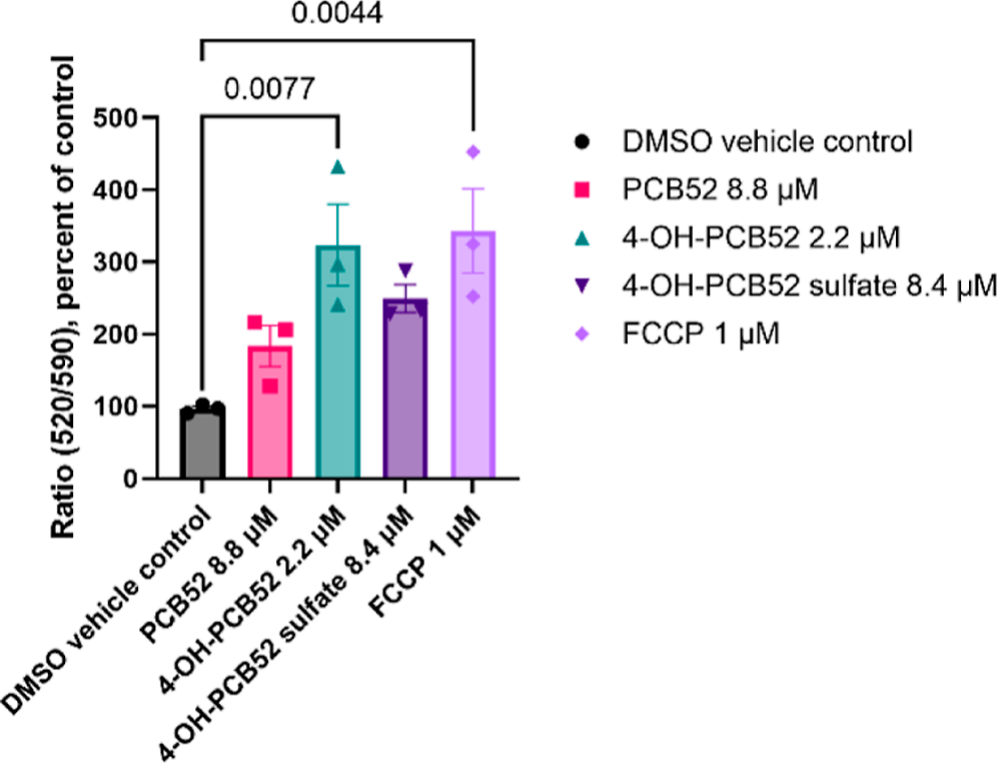
Mitochondrial membrane potential measured using a JC-10 microplate reader-based assay. C6 cells were exposed to IC_50_ concentrations of PCB52, 4-OH-PCB52, and 4-OH-PCB52 sulfate for 2 h, after which the fluorescence intensities for green monomeric (indicating loss of membrane potential) and red aggregated (indicating normal membrane potential) forms of JC-10 dye, Ex/Em 490/520 nm and Ex/Em 540/590 nm, respectively, were measured. Data are represented as the ratio of emission wavelengths of green monomeric and red aggregated forms. *n* = 3 with 4 technical replicates per “*n*”, data are represented as mean ± SEM; one-way ANOVA with post hoc Dunnett’s test was used for data analysis, *p* values <0.05 indicating statistical significance are shown in the figure.

Maintenance of mitochondrial membrane potential (ΔΨm) is necessary to produce ATP using OXPHOS.^26^ An increase or decrease in ΔΨm has been associated with various pathologies and loss of cell viability.^26^ To evaluate if PCB52, 4-OH-PCB52, or 4-OH-PCB52 sulfate alters the ΔΨm, we used the mitochondrial membrane-sensitive dye JC-10. JC-10 dye selectively enters mitochondria as a red aggregated form. JC-10 diffuses out of mitochondria as the membrane potential decreases and changes to a green monomeric form. This change can be measured using the excitation and emission wavelengths of the green and red forms of JC-10. All three compounds, PCB52, 4-OH-PCB52, and 4-OH-PCB52 sulfate, decreased ΔΨm 2 h postexposure, with 4-OH-PCB52 being the most potent (Figure 4). Carbonyl cyanide-*p*-trifluoromethoxyphenylhydrazone (FCCP) at 1 μM was used as the positive control.

### PCB52 and its Human-Relevant Metabolites Cause Distinct Structural Changes in Mitochondria

**Figure 5:**
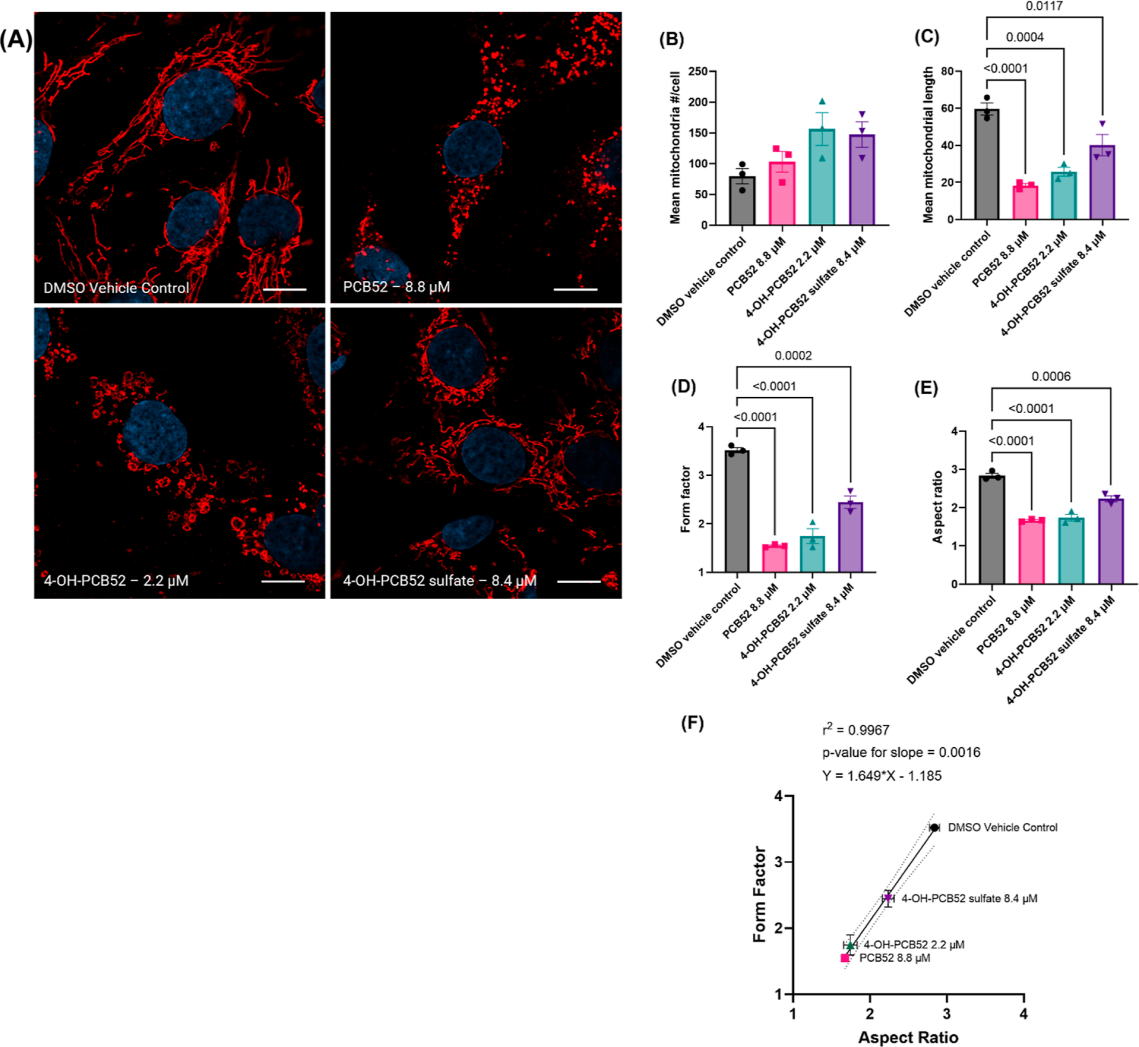
HSP60-stained C6 cells following exposure to PCB52 and its metabolites. (A) C6 cells were exposed to PCB52 and its human-relevant metabolites for 2 h and then fixed, immunostained for HSP60, a mitochondrial matrix protein, and imaged to visualize the mitochondrial structure. Data analysis of confocal images showed that PCB52 and both the metabolites (B) increased mitochondrial number and significantly decreased (C) mitochondrial length, (D) form factor, and (E) aspect ratio. (F) *XY* plot of the two independent metrics, the form factor and aspect ratio, indicating both metrics change in the same direction. *n* = 3 with 2 technical replicates per “*n*”, at least 10 images per “*n*” were analyzed (at least ∼30 astrocytes per exposure condition); scale bar, 10 μm; data were analyzed using one-way ANOVA with post hoc Dunnett’s test, *p* values <0.05 indicating statistical significance are shown in the figure.

C6 cells were exposed to PCB52, 4-OH-PCB52, and 4-OH-PCB52 sulfate for 2 h at the respective IC_50_ concentrations. Cells were then fixed, immunostained for heatshock protein 60 (HSP60), a mitochondrial matrix protein, and imaged for morphological changes. All three compounds caused distinct changes in mitochondrial morphology compared to the DMSO vehicle control: PCB52 demonstrated short, fragmented mitochondria; 4-OH-PCB52 exposure yielded small spheroid-like structures of mitochondria; and 4-OH-PCB52 sulfate exposure showed shortened mitochondria, although similar to that observed for the vehicle control (Figure 5A). Further image analysis revealed that the mean mitochondrial length was significantly reduced across all exposure conditions, accompanied by an increase in the number of mitochondria per cell (Figure 5B,C). The form factor is a measure of the complexity and branching of mitochondria as it considers the area and perimeter of mitochondrial particles and was found to be significantly reduced across all exposure conditions (Figure 5D).^27,28^ The mean aspect ratio, a ratio of mitochondrial length to width, was also significantly reduced across all exposure conditions (Figure 5E). The *XY* plot of two independent metrics, the aspect ratio and form factor, yield a straight line (Figure 5F), further showing that the morphological changes occurred for the exposure conditions used.^27^

### PCB52 and its Human-Relevant Metabolites Alter Mitochondrial Bioenergetics in C6 Cells

**Figure 6:**
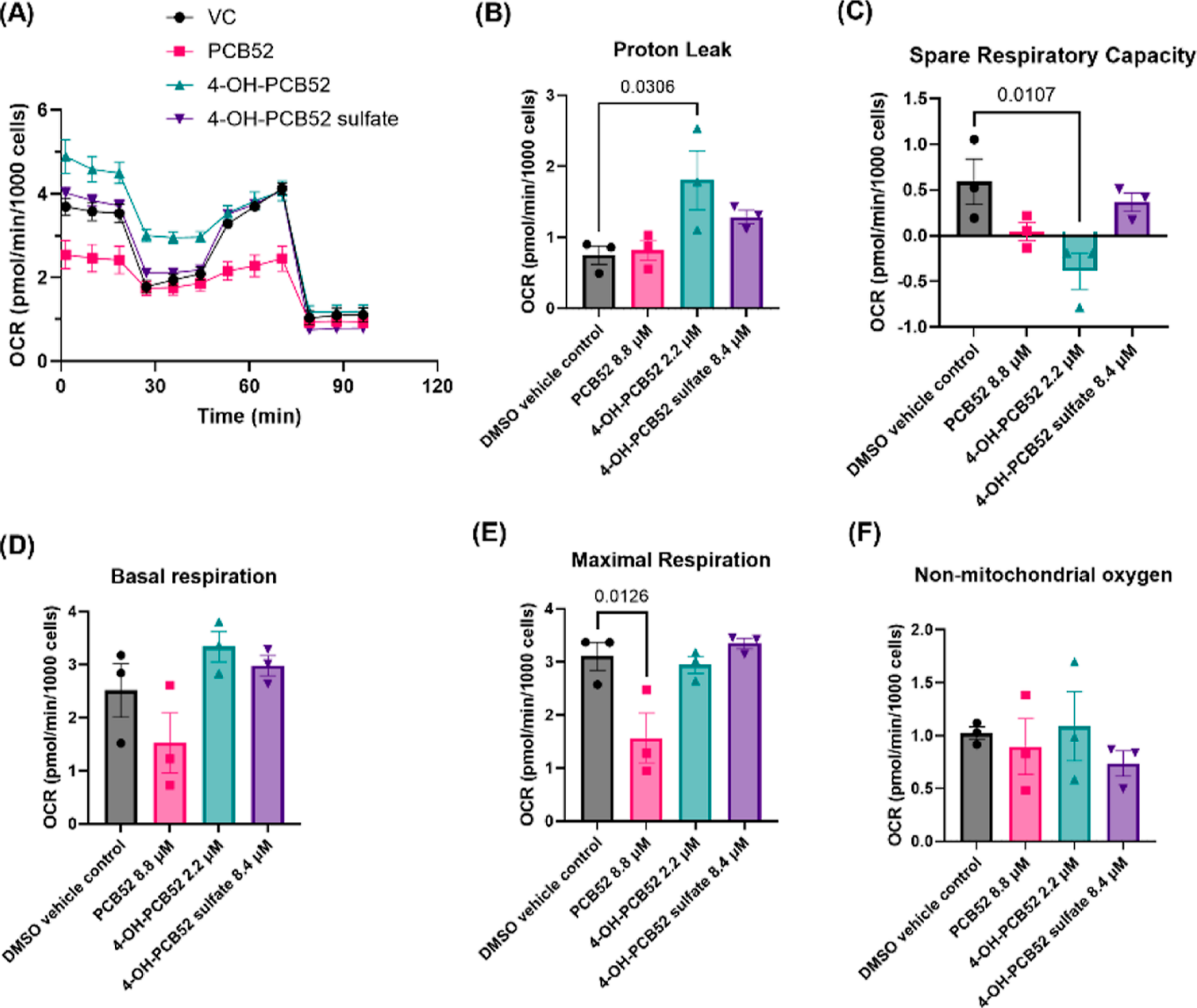
(A) OCR of C6 cells exposed to IC_50_ concentrations of PCB52 and its human-relevant metabolites after 2 h of exposure. Data analysis of the OCR showed that 4-OH-PCB52 significantly (B) increased proton leak and (C) decreased spare respiratory capacity in C6 cells. (D) Although not statistically significant, the compounds altered basal respiration of C6 cells, and (E) PCB52 significantly reduced maximal respiration. (F) No change in nonmitochondrial oxygen consumption was observed. *n* = 3 with 4 technical replicates per “*n*”, data are represented as mean ± SEM; one-way ANOVA with post hoc Dunnett’s test was used for data analysis, *p* values <0.05 indicating statistical significance are shown in the figure.

Using the Seahorse XF Cell Mito Stress Test, we evaluated mitochondrial function upon exposure to PCB52, 4-OH-PCB52, and 4-OH-PCB52 sulfate. C6 cells were exposed to IC_50_ concentrations of each of the three compounds for 2 h, after which the oxygen consumption rate (OCR) was measured following the addition of probes to interrogate facets of mitochondrial respiration. Data analysis showed that PCB52 and metabolites altered the OCR throughout the experiment (Figure 6A) and reduced spare respiratory capacity in C6 cells, with 4-OH-PCB52 being the most potent (Figure 6B–F). The exposures also produced altered basal respiration and maximal respiration compared to vehicle control (DMSO). In summary, PCB52 reduced basal respiration, while 4-OH-PCB52 increased basal respiration in C6 cells compared to the vehicle control. PCB52 decreased maximal respiration, while the metabolites did not significantly alter it. 4-OH-PCB52 also significantly increased proton leak in the C6 cells.

### PCB52 and its Human-Relevant Metabolites Induce Caspase 3/7 Activation

**Figure 7:**
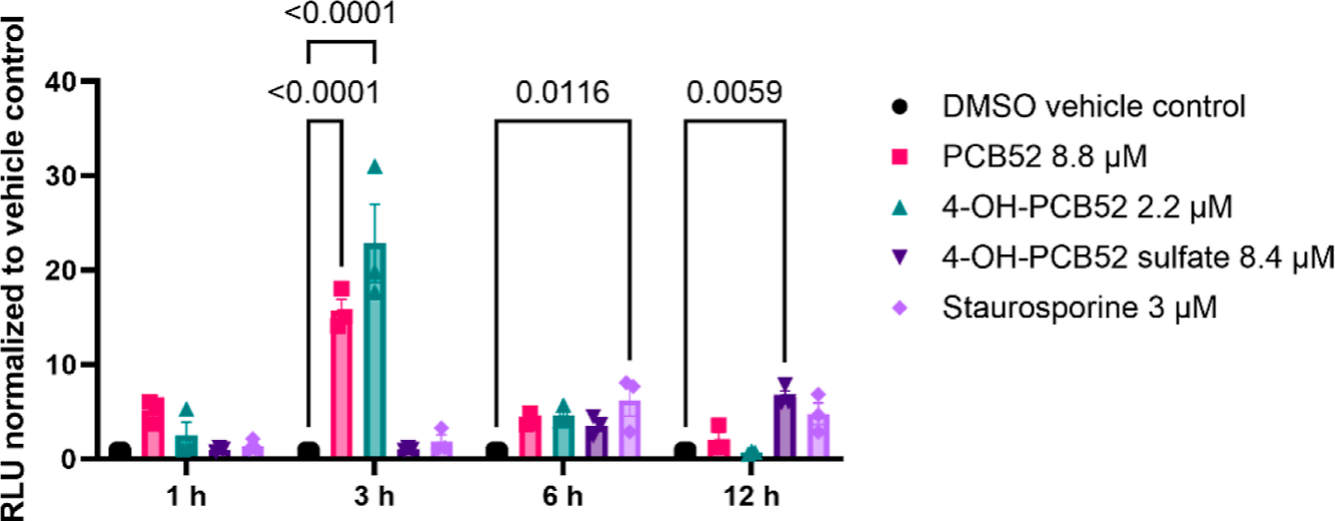
Induction of caspase 3/7 activity in C6 cells exposed to IC_50_ concentrations of PCB52 or both of its human-relevant metabolites. PCB52 and 4-OH-PCB52 significantly increased the activity of caspase 3/7 3 h postexposure, while 4-OH-PCB52 sulfate showed a late induction of the activity of caspase 3/7 in C6 cells exposed to IC_50_ concentrations of all the three compounds. *n* = 3 with 3–4 technical replicates per “*n*”, data are represented as mean relative luminescence units (RLUs) normalized to DMSO vehicle control ± SEM; one-way ANOVA with post hoc Dunnett’s test was used for data analysis, *p* values <0.05 indicating statistical significance are shown in the figure.

Caspase 3 and caspase 7 are executioner or effector caspases, which are proteolytic enzymes involved in apoptotic cell death.^29,30^ To evaluate whether caspases 3/7 are induced in C6 cells upon exposure to PCB52 or its human-relevant metabolites, we employed a Promega caspase 3/7 Glo assay. C6 cells were exposed to IC_50_ concentrations of PCB52, 4-OH-PCB52, and 4-OH-PCB52 sulfate, and the plates were read at 1, 3, 6, and 12 h postexposure. Data analysis showed that PCB52 induced caspase 3/7 activation as early as 1 h postexposure, and maximal caspase 3/7 induction was observed via PCB52 and 4-OH-PCB52 treatment 3 h postexposure. Interestingly, only at 6 h postexposure and later did 4-OH-PCB52 sulfate exposure yield significant induction compared to the negative control (Figure 7).

## Discussion

Astrocytes are abundant, complex cells with multiple cellular processes distributed within the CNS. An astrocyte can have multiple cellular processes distributed within the neuronal networks; for instance, a single protoplasmic astrocyte (a highly branched astrocyte) in the rat brain can be in contact with and regulate ∼140,000 synapses.^31^ These macroglial cells are responsible for carrying out numerous essential functions of the CNS, such as synapse pruning and regulating synaptic transmission, uptake and regulation of neurotransmitters such as glutamate, regulating the blood–brain-barrier, Ca^2+^ signaling and regulation, and responding to CNS insults such as injury or chemical exposures.^19,32^ Even though mitochondria are ubiquitously present in all cell types, their functions vary from cell to cell owing to the need for biosynthesis of metabolites, local tissue energy demands, and cellular signaling. A unique feature of astrocyte mitochondria is that they regulate various essential functions of astrocytes, such as Ca^2+^ signaling and regulation, maintaining glutamate homeostasis, and transmitophagy (transcellular mitophagy), to name a few.^8,9,33,34^

Astrocytes are primarily glycolytic cells that do not rely on OXPHOS to meet their energy needs; however, they are not deficient in OXPHOS and require functioning mitochondria.^19,21^ During aerobic glycolysis, they produce lactate from pyruvate, an energy-rich substrate shuttled to neurons to satisfy their high energy needs, through the astrocyte–neuron lactate shuttle.^19,35^ Since galactose gives a low ATP yield through glycolysis compared to glucose, the cells are pushed into mitochondrial OXPHOS in the presence of galactose and the absence of glucose.^22^ Therefore, increased cytotoxicity observed for a test compound in the presence of galactose (compared to glucose) indicates that the mitochondria are a target in observed cytotoxicity.^19,36^ We utilized galactose media as a probe to determine whether mitochondria are a target in the mechanism of toxicity for PCB52 and its metabolites in astroglial cells. We found that PCB52, 4-OH-PCB52, and 4-OH-PCB52 sulfate were more toxic to C6 cells in galactose-containing media than in glucose-containing media, indicating mitochondria to be a target for these organochlorines (Figure 2). Further, we demonstrate that human-relevant PCB52 metabolites increase mitochondria-targeted but not cellular oxidative stress (Figure 3B–D); however, pretreatment of C6 cells with the mitochondria-targeted antioxidants Mito-Tempo and Mito-Q^25^ (Supporting Information Figure S2) do not alleviate the cytotoxicity observed with PCB52 or its metabolite exposure. Such a finding indicates that mitochondrial ROS production is one of the various outcomes associated with mitochondrial damage and cellular toxicity, as demonstrated through subsequent experiments, but not a primary cause of cellular toxicity.

Mitochondria are double-membrane-bound organelles that generate a proton gradient through the proton pumps of the electron transport chain across the mitochondrial inner membrane. This proton gradient is essential for generating and maintaining the ΔΨm.^26,37^ Increase or decrease of ΔΨm for extended periods may result in cell death and pathologies.^26^ Evaluation of ΔΨm in the present study showed that 2 h of exposure to PCB52, 4-OH-PCB52, or 4-OH-PCB52 sulfate resulted in a loss of ΔΨm, with 4-OH-PCB52 being the most potent compound (Figure 4). The loss of ΔΨm also results in rapid fragmentation of mitochondria, and it has recently been shown that carbonyl cyanide *m*-chlorophenyl hydrazine (CCCP), an uncoupler of mitochondria, causes loss of ΔΨm, resulting in shortened, indented spheroids of mitochondria, in a fission-independent manner.^37^ In this study, we also report altered mitochondrial structures in C6 cells after 2 h of exposure to PCB52 or its human-relevant metabolites. Notably, with the exposure to 4-OH-PCB52, spheroid-like structures in mitochondria were observed (Figure 5A).

Mitochondria are dynamic organelles that undergo fission, fusion, and structural reorganization within cells to maintain appropriate function.^38^ Their number and structure can vary depending on the cell cycle, the physiological state of cells, or if the cells are exposed to toxicants.^39^ Mitochondria can form physically interconnected networks, sometimes called the reticulum, spanning the entire mammalian cell, which are thought to improve the transport of ions, proteins, metabolites, and mitochondrial DNA (mtDNA).^40^ Like neurons, the astrocyte cell body demonstrates a continuous network of interconnected mitochondria, whereas the astrocyte processes contain discrete, disconnected mitochondria.^41^ In our study, we report that each test compound distinctly affects the mitochondrial morphology and network, indicating their different modes of action on the mitochondria (Figure 5A). All the exposure conditions caused significantly reduced mitochondrial length and increased mitochondrial number, albeit not significantly different, suggesting that mitochondria underwent a cleaving process and became shorter in these exposed C6 cells (Figure 5B,C). Significant reduction in the form factor further illustrates that mitochondria significantly lost their complexity or branching upon exposure. The reduction of the aspect ratio indicates that they became rounder in shape. Loss of ΔΨm (Figure 4) also results in fragmentation of mitochondria, resulting in short, punctate mitochondria, which may explain the observed changes in mitochondrial morphology in this study.^37^ Mitochondrial size and mitochondrial metabolic competence are inversely correlated in adult rats.^42^ This may explain our findings suggesting that upon PCB exposure, C6 cells undergo a crisis, resulting in the shortening of mitochondria (in a fission-dependent or -independent manner) to increase their metabolic capacity and/or energy production, as a response to mitigate damage caused by PCB exposure.

We also assessed the mitochondrial metabolic activity of C6 cells using the Seahorse XF assay. Upon measuring the OCR in C6 cells after 2 h of exposure to test compounds, we found that 4-OH-PCB52 caused a significant decrease in spare respiratory capacity of C6 cells, indicating its activity as an uncoupler of mitochondria (Figure 6). Such a result corroborates the findings reported for ΔΨm and mitochondrial structures described above (Figures 4 and 5). We predict that the initial increase in basal respiration in C6 cells exposed to 4-OH-PCB52 is due to the rapid uncoupling activity, resulting in cells respiring at their maximal capacity during the 2 h exposure period; however, further evidence is necessary to establish this process.

Our findings also suggest that exposure to all three test compounds induces rapid activity of executioner caspases 3 and 7 (Figure 7). PCB52 and 4-OH-PCB52 induce caspase 3/7 activity as early as 1 h postexposure; however, microscopic examination of cells stained with Hoechst 33342 stain showed that there is no cell death observed until ∼6 h postexposure (data not shown). Loss of ΔΨm has been reported to initiate apoptotic events mediated through mitochondria,^43^ which may explain our observed data. However, it has been reported that caspases 3 and 7 play a role in the activation of astrocytes and microglia^29,44^ and downstream inflammatory processes. Further studies differentiating these processes will be necessary to elucidate the role of increased caspase 3/7 activation in astrocytes.

In the current study, we have assessed the mitochondrial toxicity of PCB52, a tetra-chlorinated biphenyl that is one of the most abundantly found lower-chlorinated biphenyl (LC-PCB) congeners in air, along with its human-relevant metabolites.^17,18^ Exposure to LC-PCBs is a public health concern as they continue to be produced even today as byproducts of chemical manufacturing processes such as the paint and pigment production processes.^45,46^ Serum levels of PCBs in populations exposed to PCBs without high dietary exposure had PCB levels ranging from undetectable to ∼650 ng/g lw.^47^ Total PCB levels in human brain samples from older adults ranged from 32 to 3050 pg/g ww, while those in neonatal brain samples ranged from undetectable to ∼2800 pg/g ww.^48^ Despite the first evidence linking PCB exposure to impaired cognitive outcomes and learning disabilities becoming known more than 5 decades ago, the mechanism of PCB-induced neurotoxic outcomes is not fully understood.^13,15^ In a recent study, PCB52 was found to localize in the brain tissue post single intraperitoneal injection across all exposure groups of 1, 10, and 100 mg/kg body weight of female Sprague–Dawley rats.^49^ In the same study, 4-OH-PCB52 was reported to be found only in the highest exposure group, only in a single animal.^49^ Another report based on an assessment of post-mortem human brain samples showed that detection frequencies of LC-PCBs were greater in the neonatal samples than in the adult brains, implying greater exposure of neonates to the ongoing inadvertent production of LC-PCBs.^48^ This is the first study that also reports hydroxylated metabolites of dichlorinated PCB from a 1 day old female donor.^48^

Mechanisms via which PCBs cause neuronal dysfunction have been explored and may include altered dopamine levels in the brain, agonism of ryanodine receptors (e.g., PCB95), and increased dendritic arborization of hippocampal neurons (e.g., PCB11).^50−52^ Dysfunction of astrocyte mitochondria has been associated with impaired Ca^2+^ handling by astrocytes, induction of inflammatory responses in astrocytes, glutamate toxicity, insufficient fatty acid metabolism, and biosynthesis of energy-rich substrates, which has been further associated with development of neurodegenerative diseases such as PD and Alzheimer’s disease (AD).^53−55^ Transmitophagy seems to be altered in AD, where AD astrocytes show increased internalization and degradation of neuronal mitochondria.^56^ Currently, there are no reports of these parameters being studied in astrocytes after PCB exposure; however, intracellular Ca^2+^ levels and signaling have been reported to be altered in neuronal cells.^50,57^

In the present study, we demonstrate, for the first time, that astrocyte mitochondria are a sensitive target of PCB52 and its human-relevant metabolites, 4-OH-PCB52 and 4-OH-PCB52 sulfate. The most toxic compound, 4-OH-PCB52, was shown to significantly reduce mitochondrial membrane potential and spare respiratory capacity and cause proton leak in mitochondria, along with inducing spheroid-like structures of mitochondria in C6 cells. These findings suggest that 4-OH-PCB52 may be acting as an uncoupler of mitochondria. Further studies will be necessary to establish the role of 4-OH-PCB52 as an uncoupler. In addition, future work exploring the effects of long-term low-dose LC-PCB exposure is necessary to better understand the effects of environmental LC-PCB exposure on the CNS.

## Materials and Methods

### PCBs

PCB52 and its hydroxylated (4-OH-PCB52) and sulfated (4-OH-PCB52 sulfate) metabolites were synthesized, authenticated, and provided by the Synthesis Core, Iowa Superfund Research Program (ISRP), as described previously.^58−62^ The current study utilizes IC_50_ concentrations of the test compounds unless stated otherwise, which we have determined in C6 cells and reported previously: PCB52 (8.8 μM), 4-OH-PCB52 (2.2 μM), and 4-OH-PCB52 (8.4 μM).^16^

### C6 Cell Culture

C6 cells (CCL-107, Lot no. 63821786) were purchased from the American Type Culture Collection (ATCC, Manassas, VA, U.S.A.) and were cultured and maintained as described.^16^ For the current study, all experiments have been conducted on C6 cells between passages 4 and 15. The C6 cells were derived from a rat glioblastoma but are similar to primary astrocytes in structure and function and have been used as an in vitro model for astrocytes.^14,63^ All experiments were performed in glucose-containing media except for the glucose-galactose assay (Figure 2) as astrocytes are primarily glycolytic cells.^21^

### Cell Viability Assay

C6 cells were seeded in Corning 24-well plates (Cat. no. 3526) at a seeding density of 6 × 10^4^ cells/well in Gibco Dulbecco’s modified Eagle medium/nutrient mixture F-12 (DMEM/F12) (Cat. no. 11320033) with 10% fetal bovine serum (FBS) (Cat. no. 26140079) and 1% penicillin–streptomycin (Pen-Strep; Cat. no. 15140122) and allowed to grow for 24 h at 37 °C in humidified conditions and 5% CO_2_. Post incubation, cells were washed with serum-free, phenol-red free Gibco DMEM (Cat. no. A1443001) supplemented with 2.5 mM l-glutamine (Cat. no. 25030081) and 0.5 mM sodium pyruvate (Cat. no. 11360070). This medium was used for exposures after further supplementation with either 17.5 mM glucose (Cat. no. G32040) or 17.5 mM galactose (Cat. no. G33000). Postwash, the C6 cells were exposed to a concentration range of 0.312 to 20 μM of each compound—PCB52, 4-OH-PCB52, and 4-OH-PCB52 sulfate, in 500 μL/well of glucose- or galactose-containing medium as described above. Such exposed cells were incubated for 24 h at 37 °C in humidified conditions and 5% CO_2_. Postincubation, the medium was aspirated, and the Alamar blue assay was performed per the manufacturer’s instructions for Invitrogen alamarBlue Cell viability reagent (Cat. no. DAL1100). Cell viability was calculated with respect to the DMSO vehicle control. Each experiment had 3 technical replicates and was performed for *n* = 3.

### ROS Detection

DCFDA assay: C6 cells were seeded in Corning black-walled 96-well plates (Cat. no. 3916) at a seeding density of 2 × 10^4^ cells/well in DMEM/F12 supplemented with 10% FBS and 1% Pen-Strep and allowed to grow for 24 h at 37 °C in humidified conditions and 5% CO_2_. Postincubation, cells were washed once in 1× phosphate-buffered saline (PBS), treated with 15 μM DCFDA reagent, and incubated at 37 °C for 45 min in the dark. Cells were washed with 1× PBS and exposed to PCB52 and its human-relevant metabolites in serum-free, phenol-red free Gibco DMEM/F12 medium (Cat. no. 21041025) with 1% Pen-Strep, referred to as the exposure medium further. The fluorescence intensity was measured every hour for up to 4 h postexposure using the BioTek Synergy HTX multimode plate reader. MitoSox and CellRox assays: C6 cells were seeded in Cellvis black-walled 24-well plates (Cat. no. P24-1.5H-N) at a seeding density of 6 × 10^4^ cells/well in DMEM/F12 supplemented with 10% FBS and 1% Pen-Strep and allowed to grow for 24 h at 37 °C in humidified conditions and 5% CO_2_. Postincubation, the cells were washed. Cells were treated with MitoSox Red (Cat. no. M36008) and CellRox Green (Cat. no. C10444) separately per the manufacturer’s instructions. MitoSox Red was used at a concentration of 2 μM, and CellRox Green was used at a concentration of 5 μM. Postincubation, cells were washed and exposed to IC_50_ concentrations of PCB52, 4-OH-PCB52, and 4-OH-PCB52 sulfate in the exposure medium. Antimycin A, 2.5 μM (Cat. no. A8674) and Menadione, 15 μM (Cat. no. 15950) were used as positive controls for MitoSox Red and CellRox Green assays, respectively. Cells were then treated with NucBlue Live cell stain for 10 min to stain the nuclei, after which cells were imaged periodically using a 20× objective on the EVOS FL Auto II microscope. Each experiment was performed for *n* = 4. At least 5 images per “*n*” were analyzed. Image analysis: Images were analyzed using Fiji (version 1.52 or higher), open-source image processing software, and fluorescence intensity was quantified using a macro developed by Stefan Strack at the University of Iowa. The macro used for image analysis, Densitometry.txt, is openly available at the following GitHub repository: https://github.com/ststrack/Strack-Lab-software.

### Mitochondrial Structure Assessment

C6 cells were seeded on Nunc Lab-Tek 4-well chambered coverglass with nonremovable wells (Cat. no. 155383) at a density of 2 × 10^4^ cells/well in DMEM/F12 with 10% FBS and 1% Pen-Strep. After 24 h incubation at 37 °C in humidified conditions and 5% CO_2_, cells were washed once with serum-free phenol-red-free DMEM/F12 medium (exposure medium) and then exposed to the respective IC_50_ concentrations of PCB52, 4-OH-PCB52, and 4-OH-PCB52 sulfate in the exposure medium. Cells were incubated for 2 h at 37 °C under humidified conditions and 5% CO_2_. Cell fixation: Postincubation, the medium was aspirated, and cells were washed once with 200 μL/well of 1× PBS. Cells were then fixed in 200 μL of 4% paraformaldehyde in PBS (Cat. no. AAJ61899-AP) per well at room temperature (RT) for 15 min. Cell permeabilization: The fixation solution was aspirated, and cells were washed twice with 200 μL of PBS. Cells were permeabilized with 200 μL of 0.5% Triton-X in PBS per well at RT for 5 min. Blocking: Permeabilization solution was aspirated, and cells were washed twice with 200 μL/well of 1× PBS. Blocking was done with 200 μL/well of blocking solution (1% goat serum and 1% donkey serum in PBS) at RT on a shaker for 1 h. Primary antibody staining: Blocking solution was aspirated, and cells were washed twice with 200 μL/well of 1× PBS. The Proteintech anti-HSP60 antibody (Cat. no. 66041-1-Ig), provided by Dr. Strack, was diluted at a ratio of 1:500 in blocking solution. 150 μL of primary antibody solution was added to each well, and cells were incubated overnight at 4 °C. Secondary antibody staining: Primary antibody solution was aspirated, and cells were washed thrice with 200 μL/well of 1× PBS. Goat antimouse Alexa 647 (Cat. no. **A-21235**) was prepared at a dilution of 1:500 in the blocking solution. 150 μL of secondary antibody solution was added to each well, and cells were incubated for 2 h at RT, wrapped in aluminum foil. Postincubation, the secondary antibody solution was aspirated, and cells were washed thrice with 200 μL/well of 1× PBS. The chambered coverglass was kept on a shaker for 10 min between each wash. During the incubation after the second wash, Hoechst 33342 was added to the cells to stain nuclei. After washing thrice, 200 μL/well of 1× PBS was added to each well, and the coverglass was wrapped in aluminum foil and stored at 4 °C until imaging analysis. Confocal imaging: HSP60 stained C6 cells were imaged using an oil objective of 63× on a Zeiss LSM-980 at the central microscopy research facility (CMRF) at the University of Iowa. Image analysis: Images were analyzed using Fiji (version 1.52 or higher), open-source image processing software, and mitochondrial morphometry was done using a macro developed by Dr. Stefan Strack at the University of Iowa. The macro used for image analysis, Morphometry.txt, is openly available at the following GitHub repository: https://github.com/ststrack/Strack-Lab-software. Each experiment had 2 technical replicates and was performed for *n* = 3. At least 10 cells from each experiment (∼30 cells total) were used for data analysis.

### Mitochondrial Membrane Potential Assay

C6 cells were seeded in Corning black-walled 96-well plates (Cat. no. 3916) at a density of 2 × 10^4^ cells/well. After 24 h of incubation at 37 °C in humidified conditions and 5% CO_2_, cells were exposed for 2 h to the respective IC_50_ concentrations of PCB52, 4-OH-PCB52, and 4-OH-PCB52 sulfate in the exposure medium. DMSO was the vehicle control, and 1 μM FCCP was the positive control. After 2 h of incubation, the medium was aspirated, and the manufacturer’s instructions for the Abcam mitochondrial membrane potential assay kit (Cat. no. ab112134) were followed. Fluorescence intensity was measured using a BioTek Synergy HTX multimode plate reader per the manufacturer’s instructions. Data were analyzed with respect to the vehicle control and represented as the ratio of emission wavelengths of the green monomeric form and orange aggregated form of JC-10 dye (520/590). Each experiment has 4 technical replicates and was performed for *n* = 3.

### Mitochondrial Stress Test Using a Seahorse Analyzer

C6 cells were seeded in XFp24 plates at a seeding density of 2 × 10^4^ cells/well in DMEM/F12 supplemented with 10% FBS and 1% Pen-Strep and allowed to grow for 24 h at 37 °C in humidified conditions and 5% CO_2_. Postincubation, cells were washed once with the exposure medium and exposed to IC_50_ concentrations of PCB52, 4-OH-PCB52, and 4-OH-PCB52 sulfate in the exposure medium. Cells were then incubated for 1 h at 37 °C under humidified conditions and 5% CO_2_. The plate was transferred to a CO_2_-free incubator for the next hour of incubation for degassing, during which bright-field images of all the wells of the plate were taken using a BioTek Cytation I cell imaging multimode reader housed in the Metabolic Phenotyping core at the University of Iowa. After 2 h of incubation, cells were washed twice with Seahorse XF DMEM supplemented with 2.5 mM l-glutamine and 0.5 mM sodium pyruvate, and a final volume of 500 μL/well of this media was added to the cells. The Seahorse assay was run via an Agilent Seahorse XF24 analyzer per the manufacturer’s instructions. Briefly, a cycle of wait–mix–measure of 2–3–2 min was used, and three measurements of OCR per measurement cycle were recorded. Three injections at final concentrations of 1 μM Oligomycin A (Port A), 1 μM FCCP (Port B), and 1 μM Rotenone/Antimycin A (Port C) were used. The final injection through port C also had Hoechst 33342 for nuclear staining of C6 cells. Post-assay, fluorescence images of each well were captured, and the cell count was recorded using a BioTek Cytation I cell imaging multimode reader. The measured OCR was normalized to this cell count. Data analysis was performed per the manufacturer’s instructions. Each experiment had 4 technical replicates and was performed for at least *n* = 3.

### Induction of Caspase 3/7 Activity

To evaluate if PCB52 and its human-relevant metabolites induce the activity of executioner caspases 3 and 7, we used the Promega Caspase 3/7 Glo assay (Cat. no. G8091). C6 cells were seeded in Greiner Bio-One 96-well white-walled microplates (Cat. no. 655088) at a seeding density of 20 × 10^4^ cells/well in DMEM/F12 supplemented with 10% FBS and 1% Pen-Strep and allowed to grow for 24 h at 37 °C in humidified conditions and 5% CO_2_. Postincubation, cells were treated with the assay reagents per the manufacturer’s instructions and exposed to IC_50_ concentrations of PCB52, 4-OH-PCB52, and 4-OH-PCB52 sulfate in the exposure medium. The microplate was read using a BioTek Synergy HTX multimode plate reader for luminescence intensity at 1, 3, 6, and 12 h postexposure. Data were normalized to the DMSO vehicle control. 3 μM Staurosporine (Cat. no. 569396) was the positive control. Each experiment had 3–4 technical replicates and was performed for an *n* = 3.

## Data Availability

Data for all experiments were analyzed by GraphPad Prism version 8.3.0 and above for Windows (GraphPad Software, San Diego, CA, U.S.A.). The data that support the findings presented in this manuscript are available on Iowa Research Online repository at https://doi.org/10.25820/data.007101.^64^

## Supporting Information Available

Cell viability of C6 cells exposed to increasing concentrations of PCB52, 4-OH-PCB52, and 4-OH-PCB52 sulfate and cell viability of C6 cells exposed to IC_50_ concentrations of PCB52, 4-OH-PCB52, and 4-OH-PCB52 sulfate, pretreated with mitochondria-targeted antioxidants (PDF)

## Abbreviations

AD: Alzheimer’s disease
ADHD: attention deficit hyperactivity disorder
ASD: autism spectrum disorder
ATCC: American Type Culture Collection
ATP: adenosine triphosphate
CCCP: carbonyl cyanide *m*-chlorophenyl hydrazine
CMRF: central microscopy research facility
CNS: central nervous system
DCFDA: 2′,7′-dichlorofluorescin diacetate
DMEM/F12: Dulbecco’s modified Eagle medium/nutrient mixture F-12
FBS: fetal bovine serum
FCCP: carbonyl cyanide-*p*-trifluoromethoxyphenylhydrazone
HSP60: heatshock protein 60
IC_50_: inhibitory concentration 50
ISRP: Iowa Superfund Research Program
LC-PCBs: lower-chlorinated biphenyls
mtDNA: mitochondrial DNA
OCR: oxygen consumption rate
OXPHOS: oxidative phosphorylation
PCBs: polychlorinated biphenyls
PD: Parkinson’s disease
Pen-Strep: penicillin–streptomycin
ROS: reactive oxygen species
ΔΨm: mitochondrial membrane potential
