# Supplementary material for: Astrocyte Mitochondria Are a Sensitive Target of PCB52 and its Human-Relevant Metabolites
Source: ACS Chem Neurosci. 2024 Jul 2;15(15):2729–40. doi: 10.1021/acschemneuro.4c00116 (PMC11311133; doi:10.1021/acschemneuro.4c00116)
Supplement: Supplementary file 1 — cn4c00116_si_001.pdf [file cn4c00116_si_001.pdf]

Supporting Information

Astrocyte Mitochondria are a Sensitive Target of PCB52 and its Human-relevant Metabolites.

Neha Paranjape[1,2], Stefan Strack[2], Hans-Joachim Lehmler[2], Jonathan A. Doorn[1, *]

[1]Department of Pharmaceutical Sciences & Experimental Therapeutics, College of Pharmacy, University of Iowa, Iowa City, IA, USA
[2]Department of Neuroscience and Pharmacology, University of Iowa Carver College of Medicine, Iowa City, IA, USA
[3]Department of Occupational and Environmental Health, College of Public Health, University of Iowa, Iowa City, IA, USA

*Corresponding Author:

Jonathan A. Doorn
Professor and Chair
Department of Pharmaceutical Sciences & Experimental Therapeutics
College of Pharmacy, 536 CPB
The University of Iowa
180 S. Grand Ave.
Iowa City, IA 52242 USA

Email: jonathan-doorn@uiowa.edu
TEL: 319-335-8834

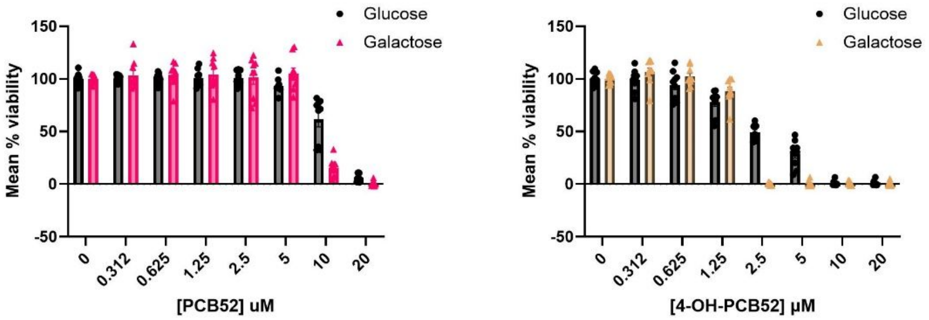

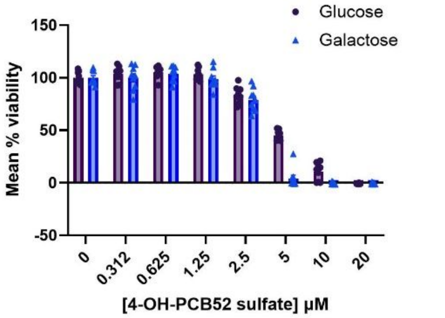

	IC_{50} Glucose (µM)	IC_{50} Galactose (µM)
PCB52	12.7	9.1
4-OH-PCB52	2.6	1.4
4-OH-PCB52 sulfate	4.5	3.1

Supplementary Figure S1: Alamar Blue cell viability assay of C6 cells exposed to varying concentrations of PCB52 and its human-relevant metabolites for 24 h in glucose- or galactose-containing media. Data represented as percent viability of untreated control ± SEM, n=3.

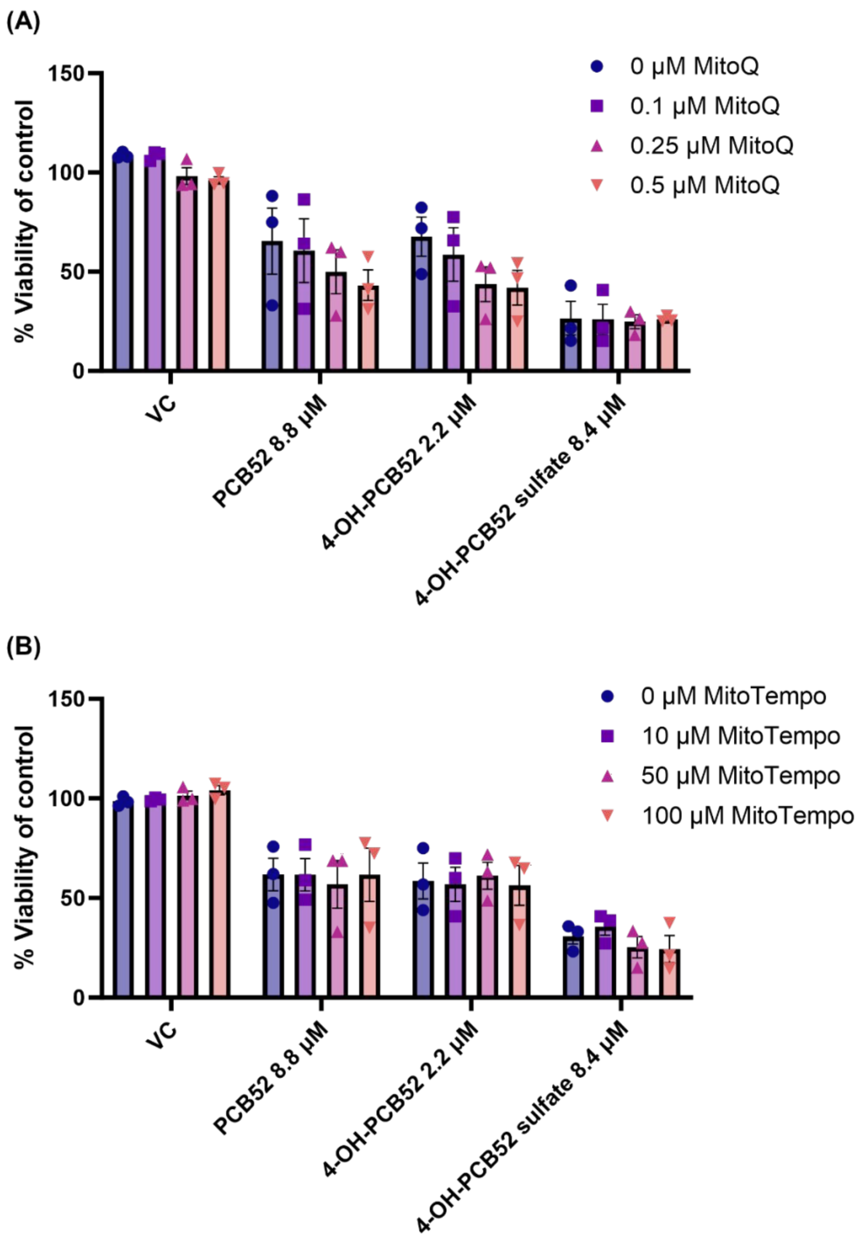

Supplementary Figure S2: Alamar Blue cell viability assay of C6 cells exposed to IC_{50} concentrations of PCB52 and its human-relevant metabolites pretreated for 2 h with mitochondria-targeted antioxidants (A) MitoQ or (B) MitoTempo. Data represented as percent viability of untreated control ± SEM, n=3 with 2 technical replicates per 'n'.
